# Preparation and Properties of Egg White Dual Cross-Linked Hydrogel with Potential Application for Bone Tissue Engineering

**DOI:** 10.3390/polym14235116

**Published:** 2022-11-24

**Authors:** Bingchao Duan, Minghui Yang, Quanchao Chao, Lan Wang, Lingli Zhang, Mengxing Gou, Yuling Li, Congjun Liu, Kui Lu

**Affiliations:** School of Chemical Engineering and Food Science, Zhengzhou University of Technology, Zhengzhou 450044, China

**Keywords:** egg white hydrogel, dual cross-linking, metal ions, secondary structure, biocompatibility

## Abstract

In this study, an egg white dual cross-linked hydrogel was developed based on the principle that the external stimulus can denature proteins and cause them to aggregate, forming hydrogel. The sodium hydroxide was used to induce gelation of the egg white protein, subsequently introducing calcium ions to cross-link with protein chains, thereby producing a dual cross-linked hydrogel. The characteristics of the dual cross-linked hydrogels—including the secondary structure, stability, microstructure, swelling performance, texture properties, and biosafety—were investigated to determine the effects of calcium ion on the egg white hydrogel (EWG) and evaluate the potential application in the field of tissue engineering. Results showed that calcium ions could change the β-sheet content of the protein in EWG after soaking it in different concentrations of CaCl_2_ solution, leading to changes in the hydrogen bonds and the secondary structure of polypeptide chains. It was confirmed that calcium ions promoted the secondary cross-linking of the protein chain, which facilitated polypeptide folding and aggregation, resulting in enhanced stability of the egg white dual cross-linked hydrogel. Furthermore, the swelling capacity of the EWG decreased with increasing concentration of calcium ions, and the texture properties including hardness, cohesiveness and springiness of the hydrogels were improved. In addition, the calcium cross-linked EWG hydrogels exhibited biocompatibility and cell-surface adhesion in vitro. Hence, this work develops a versatile strategy to fabricate dual cross-linked protein hydrogel with biosafety and cell-surface adhesion, and both the strategy and calcium-egg white cross-linked hydrogels have potential for use in bone tissue engineering.

## 1. Introduction

Natural macromolecules are a kind of polymer, existing in plants and animals, including the human body. They include polysaccharides, peptides, polynucleotides, and polyesters [1]. Natural polymers are an abundant resource for applications in the food and medical industries, due to that they have many advantages such as good biocompatibility, biodegradability, non-toxicity, and sustainability [2,3]. Hydrogel is a kind of three-dimensional network material. Hydrogels prepared from natural polymers should inherit the advantages of natural macromolecules, such as biocompatibility and biodegradability [4]. Thus, they are attracting attention particularly in biotechnology for uses such as drug delivery [5], biological sensing [6], wound dressing [7], and desalination [8,9].

Protein is a kind of natural polymer, abundant in nature, and with great prospects in polymer research [10,11]. In the field of polymer material science, the development of protein hydrogels is an important direction of current research [12,13,14]. Protein hydrogels retain a three-dimensional network structure similar to the extracellular matrix of animal tissue, featuring high water content. Hydrogels based on the proteins collagen [15], fibrin [16], elastin [17], and silk [18] have exhibited superior characteristics as compared to synthetic polymer-based hydrogels, such as biocompatibility, biodegradability, and low immunogenicity. Thus, these natural hydrogels are being extensively used for tissue engineering of bone and cartilage and in other biomedical application [19,20,21,22].

Egg white protein has a variety of functional characteristics, such as gelation, water holding capacity, foaming and emulsification. It is important in food manufacturing because it can improve the functionality, texture and flavor of food products [23]. Under heating, freezing, high pressure, extreme acid and alkali, ions, enzyme and other treatments, egg white protein is able to coagulate and form hydrogel with a three-dimensional network structure, which can strongly affect the structures, senses and flavors of egg white protein products [24,25]. The formation of protein hydrogels can be attributed to the formation of hydrogen bonds, disulfide bonds and electrostatic interaction, which induce the aggregation of protein molecules [26]. Proteins undergo denaturation in response to certain external stimuli (e.g., physical factors such as heating, ultraviolet light and pressure or pH, metal ions), and denaturation is one of the important mechanisms in the formation of protein hydrogels. The gelation of egg white protein involves multiple processes, including denaturation, aggregation and formation of gel network, and the gelling properties mainly depend on the medium conditions such as pH, ionic strength and salt type [27]. The interaction and the molecular conformation of protein chains will change during protein hydrogel formation. Studies have revealed that metal ions are able to impact the intermolecular and intramolecular interactions of protein chains and the conformation of protein molecules, further affecting the characteristics of protein gels [28,29].

**Figure 1:**
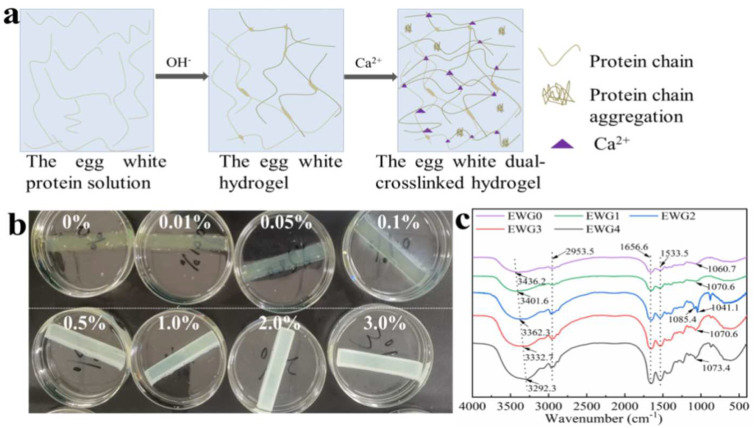
(**a**) Schematic of the EWG hydrogel preparation process; (**b**) photos of the hydrogel immersed in different concentrations of CaCl_2_ solution. The number represents the concentration of CaCl_2_ solution; (**c**) FT−IR spectra of the hydrogels.

In recent years, hydrogel materials have been extensively used in bone tissue engineering [30,31]; however, the application of protein hydrogels in bone tissue restoration and bone scaffold are severely limited due to their poor mechanical property [32]. Currently, tough and strong hydrogels are achieved by building dual network structures, compositing inorganic nanoparticles and introducing conductive materials and fibrous networks, improving the mechanical properties and the bone tissue repair ability of hydrogels [32]. Traditional hydrogels are fabricated through a single cross-linked mode, resulting in the lack of the energy dissipation pathways. Meanwhile, the dual crosslinking can improve the intermolecular interaction and cross-linking density in the hydrogel, providing an effective way to dissipate energy and increase the mechanical strength [33]. In this study, we prepared egg white hydrogel (EWG) according to the principle that proteins could be denatured by strong alkali, subsequently introducing calcium ions to induce aggregation and cross-linking with protein chains, thereby producing a dual cross-linked hydrogel (Figure 1a). Moreover, the secondary structure, stability, microstructure, swelling performance, texture and biocompatibility of the obtained hydrogels were investigated to research the effect of calcium ion on EWG.

## 2. Materials and Methods

### 2.1. Materials

Sodium hydroxide (NaOH) and calcium chloride (CaCl_2_) were provided by Aladdin (Shanghai, China). Fresh eggs were purchased from a supermarket. The egg white was carefully separated from the yolk and then kept in a sealed chamber at 4 °C until further use. The Dulbecco’s Modified Eagle’s Medium (DMEM; glutamine, high glucose), fetal bovine serum (FBS), 3-[4,5-dimethylthiazol-2-yl]-2,5-diphenyl tetrazolium bromide (MTT) and the cell staining agent (Calcein AM and PI) were obtained from Sangon Biotech (Shanghai) Co., Ltd., Shanghai, China. All other chemicals were of analytical grade and used without further purification.

### 2.2. Preparation of the Egg White Hydrogel

NaOH solution (25 mg/mL) was added dropwise into the egg white (3:7 volume ratio) at room temperature with gentle stirring, then the solution was transferred to a mold (60 mm × 15 mm × 40 mm) and kept stationary 4 °C to release any air bubble present in the solution. After gelling, the egg white hydrogel was obtained and washed thoroughly with pure water to remove the residual sodium hydroxide.

CaCl_2_ was dissolved in deionized water to obtain the sample solution at different concentrations. The egg white hydrogel samples (the samples were prepared in the size of 15 mm × 15 mm × 50 mm with a knife) were immersed in CaCl_2_ solution (0.1%, 0.5%, 1%, 2%, 3% *w*/*v*), respectively, for 2 h at room temperature. After soaking, the hydrogel samples were taken and washed with pure water to remove the calcium chloride on the surface. The hydrogel samples soaked by pure water and CaCl_2_ solution by 0.1%, 0.5%, 1% and 2% (*w*/*v*) were coded as EWG0, EWG1, EWG2, EWG3 and EWG4, respectively.

### 2.3. Characterization

The wet hydrogels were frozen in liquid nitrogen and snapped immediately, then freeze-dried using a vacuum freezing dryer (LEG-10C, Sihuan Furui Technology Development Co., Ltd., Hong Kong, China). The freeze-drying conditions were as follows: vacuum: 1Pa; cryo-temperature: −70 °C; material temperature: −50 °C; duration of drying: 24 h. The fracture sections of the freeze-dried samples were sputtered with gold for scanning electron microscopy (SEM, Zeiss, SIGMA, Roedermark, Germany) analysis.

The hydrogel samples were cut into particle-like size after being freeze-dried and vacuum-dried for 2 h at 60 °C. Then samples were transfer to a mortar and grind to a fine powder for measurements. The structural changes of the hydrogels were characterized by Fourier transform infrared spectroscopy (FT−IR, Spectrum3, Perkin Elmer, Waltham, MA, USA), X-ray Diffraction Analysis (XRD, Regaku ultima IV, Japan). The data of amide I band (1700–1600 cm^−1^) of FT-IR spectrum were analyzed by PeakFit software.

### 2.4. Swelling Tests 

The thermal stability of the hydrogels was investigated by thermogravimetric analysis (TG, Discovery TGA 550, New Castle, DE, USA). The analysis was performed from room temperature to 800 °C with a heating rate of 15 °C/min in air atmosphere.

The swelling ratios of the hydrogels in pure water were tested through the gravimetric method. The freeze-dried hydrogels were immersed into pure water at 37 °C for modelling the body temperatures. Then the water in the surface of the hydrogels was gently wiped, and the weight of the samples was registered at predefined period. The swelling ratio (SR) was calculated as SR = (Ws − Wd)/Wd, where the Ws and Wd are the weight of the swollen and dried hydrogel, respectively. Similarly, the porosity ((Ws − Wd)/Ws) of different hydrogels after achieving swelling equilibrium was determined by using the identical measurement method of the mass swelling ratio [34].

### 2.5. Texture Tests

Texture analysis was performed using a texture analyzer (TA, BROOKFIELD CT3, USA) at room temperature. The hydrogel samples were prepared in the size of 10 mm × 10 mm × 15 mm with a knife, and the cylindrical probe TA4/1000 cylinder was selected for measurement. The clipped samples were compressed twice at 1 mm/s to 50% of their original height. The results were calculated with Texture Expert version 1.22 (Stable Micro Systems, Surrey, UK). All of these steps were performed six times.

### 2.6. Cell Experiments

HEK293 cells (human embryonic kidney-293 cells) were obtained from the China Center for Typical Culture Collection and cultured at 37 °C in a 5% CO_2_ incubator. The culture medium containing 89.20% DMEM with 98 μg/mL penicillin/Streptomycin, 9.80% fetal bovine serum was changed every 2 days. 

The cytotoxicity of the hydrogels was determined via an MTT assay. The hydrogel samples (100 mg) were sterilized under a UV lamp for 24 h, and then placed in DMEM containing 9.80% fetal bovine serum for 24 h at 37 °C to prepare the hydrogel extract solution. Thereafter, the resulting extracts were filtered using a 0.22-mm syringe for MTT tests. HEK293 cells (5 × 10^3^ cells/well) were incubated in 96-wells plates for 12 h, and the extract solution of different hydrogels was added for 24 h incubation. Then the medium was aspirated and 10 μL of MTT (5 mg/mL) was added to each well for another 4-h incubation. After the culture medium was removed, 200 μL of DMSO was added in each well. After gently shaking for few minutes, the plates were carried out to measure the absorbance at 492 nm using a microplate reader (SparkTM 10M, Tecan). The cell viability was calculated based on the equation: Cell viability (%) = (the absorbance of sample-treated group/the absorbance of PBS-treated group) × 100%.

The live/dead cell staining assays were performed to evaluate the cell adhesion on the hydrogel surfaces and the biocompatibility. The hydrogel EWG4 sample with 10 mm diameter and a thickness of about 0.1 cm was sterilized under a UV lamp for 24 h, and then transferred to the bottom of 24-well plastic culture plates. HEK293 cells were seed to 24-well plastic culture plates on the sample (5 × 10^4^ cells/well) for 48 h incubation. Then, the samples were washed by physiological saline and stained by the cell staining agent (Calcein AM and PI) for 30 min at 37 °C. Thereafter, the samples were washed again for three times with physiological saline and then observed using a fluorescence microscope (Olympus BX51, Olympus Corporation, Tokyo, Japan).

### 2.7. Statistical Analysis

All measurements were repeated at least three times independently and expressed as mean ± standard deviation (SD). All values reported in this study are expressed as mean ± standard deviation, and *p* < 0.05 (*), *p* < 0.01 (**) and *p* < 0.001 (***) signify significant and extremely significant differences, respectively.

## 3. Results and Discussion

### 3.1. Preparation and Morphology Analysis of the EWG Hydrogel

There are a variety of proteins in fresh egg white solution. In the liquid state, these proteins are stabilized via electrostatic interaction, hydrogen bonds and thiol ester bonds between protein chains. However, when heated or exposed to high pressure, alkali or acid, these chains are induced to unfold and rearrange into various forms. In this work, the egg white hydrogel was prepared by alkali induction and it was transparent, light yellow, smooth and ductile. Then, the egg white hydrogel was soaked in calcium chloride solution with different concentrations in order to induce secondary cross-linking between the calcium ions and the egg white hydrogel through the interaction of calcium ions with the particular amino acids on the polypeptide chain. As shown in Figure 1b, the hydrogel samples remained solid without breaking or decomposition after soaking in calcium chloride solution for 2 h. With increasing concentration, more calcium ions gradually infiltrated into the hydrogel, leading to deeper color and decreased transparency of the hydrogels. 

### 3.2. FT−IR Analysis

The structural characteristics and conformational changes of the hydrogels were evaluated by Fourier transform infrared spectroscopy (FT−IR). As shown in Figure 1c, the absorption peaks at ~3350 and 2953 cm^−1^ were assigned to the stretching vibrations of N–H and O–H and of intermolecular hydrogen bonds, respectively [35]. With the increase of calcium concentration, the absorption band at 3436.2 cm^−1^ gradually shifted to 3292.3 cm^−1^ and the intensity of peaks at 2953 cm^−1^ increased significantly, suggesting that the introduction of calcium ions affected the hydrogen bonds between the amino and hydroxyl groups in the polypeptide chain and the protein molecular chain. The typical absorption peaks at 1656 and 1533 cm^−1^ were related to the amide I (C=O stretching) and amide II (N–H bending) modes of the protein chain structure, respectively [36]. The intensity changes of the peaks at 1533.5 cm^−1^ indicated that the amino and hydrogen bond in the protein molecular were impacted by calcium ions. Previous studies [35] have demonstrated that the amide I band (1700–1600 cm^−1^) is the most characteristic spectral region related to the secondary structure of proteins and polypeptides, and the secondary structures and conformational changes could be investigated by quantitative analysis of the amide I band. Therefore, the absorption peak of this region was used to quantitatively calculate the specific proportion of each secondary structure (α-helices 1650–1658 cm^−1^, β-sheets 1640–1610 cm^−1^, β-turns 1700–1660 cm^−1^, random coils 1650–1640 cm^−1^) [35,37]. The results showed (Table 1 and Figure S1) that the β-sheets content of the hydrogel increased significantly as calcium ion concentration increased up to 1%, and then decreased at higher calcium concentrations. It had been indicated that the structure of β-sheets is prone to protein aggregation and particularly important for the hydrogel formation and stability [37]. The interaction of calcium ions and protein molecules could facilitate the polypeptides to fold the β-sheets; however, the cross-linking between calcium ion and protein chains along with the increasing of calcium ion concentration was dominated and disturbed gradually the hydrogen bonding in β-sheets structures of the protein, leading to the reduce of the β-sheets content in the hydrogels soaking with high concentrations of CaCl_2_ solution. Meanwhile, the original conformation of EWG0 was changed after introducing calcium ion, resulting in the decrease of β-turns content and the increase of random coil structure. In addition, the changes of the band at 1030–1090 cm^−1^ assigned to C–O stretching vibration indicated that the structure of the polypeptide chain was changed by the calcium ions. Together, the results suggested that calcium ions were able to interact with the polypeptides and change secondary structure of the protein in the hydrogel.

### 3.3. XRD Analysis and Thermal Stability

**Figure 2:**
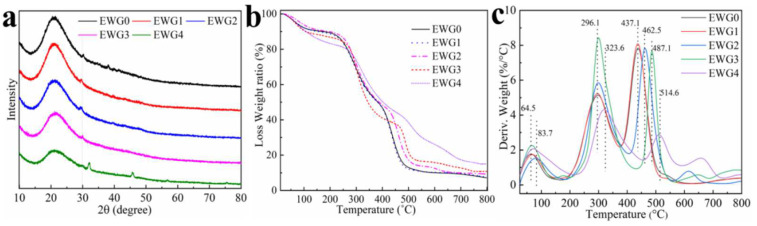
XRD patterns (**a**), TG curves (**b**) and DTG patterns (**c**) of hydrogels.

To further study the influence of calcium ions on hydrogel structure, the hydrogels were characterized by XRD, TG and DTG. The XRD spectra of the EWG0, EWG1, EWG2, EWG3 and EWG4 are shown in Figure 2a. The hydrogels exhibited distinct peaks at 2θ = 20°, which was indicated to the β-sheets secondary structure of the egg white protein. The XRD diffraction intensity of the EWG1 (5885) and EWG2 (5713) was enhanced in comparison with that of EWG0 (5233), and then the intensity decreased along with the increase in the concentration of calcium ions (EWG3:4378; EWG4:3096), confirming that the β-sheets structure in the crystalline region of the protein was disturbed by the interaction of protein with calcium ions [38,39], which was consistent with the FT−IR results.

TG and DTG analyses were applied to investigate the thermal properties of the hydrogels. The TG curves (Figure 2b) revealed that the weight loss of EWG4 (17.3%) was higher than that of EWG0 (9.8%), and the temperature corresponding to the endothermic peak on the DTG curve of the hydrogels cross-linked by calcium ions (EWG1, EWG2, EWG3 and EWG4) (Figure 2c) was increased as compared with EWG0, indicating the water retention capacity and thermal stability of the hydrogel was enhanced after interacting with calcium ion. The second stage of weight loss was mainly due to the breaking of unstable non-covalent bonds of the protein chains and the covalent bonds of the small molecules in the protein backbone. As more calcium ions were added, the hydrogel exhibited an increased degradation temperature and decreased weight loss rate. Meanwhile, the weight loss decreased from 50.767% (EWG0) to 43.926% (EWG4), which was indicative of enhanced thermal stability. The explanation for this pattern is that the interaction of calcium ions with protein promoted secondary cross-linking of the protein chain and increased hydrogel cross-linking density, which could effectively inhibit heat conduction, thereby hindering thermal degradation of protein skeleton, and enhancing the thermal stability of the hydrogel [40,41]. In summary, the results indicated that the dual cross-linked structure involving the calcium ion formed a heat-stable system, which translated into improved stability of the hydrogel.

### 3.4. Microscopic Examination

**Figure 3:**
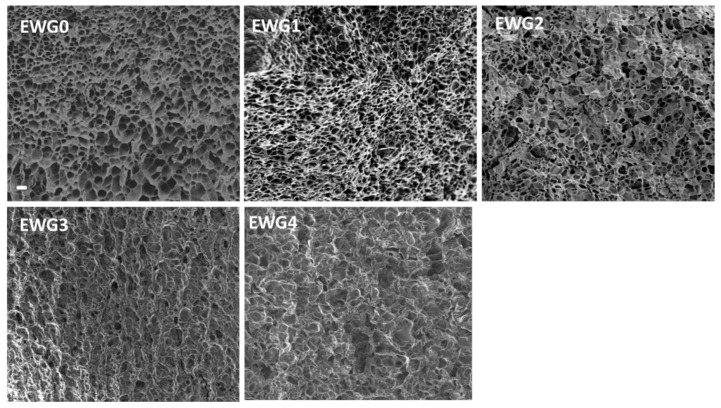
SEM images of the hydrogels. The scale bar is 10 µm.

SEM was used to study the microstructure changes of the hydrogels. As shown in Figure 3, EWG0 exhibited a loose and homogeneous three-dimensional structure with lots of pores. It had been reported that protein molecule chains were able to unfold and rearrange themselves to form an ordered three-dimensional protein network under strong alkaline condition [42]. After soaking in calcium chloride, the hydrogels exhibited inhomogeneous and rough microstructure with the disordered porous structure. When the calcium ion concentration reached 1.0% and 2.0%, the porous structure of EWG3 and EWG4 was significantly reduced, accompanied by the appearance of rough, flat edges and coarse fibers (Figure S2). The results suggested that the interaction with calcium cations induced the secondary crosslinking of the protein chains and improved the degree of crosslinking, leading to the formation of more compact microstructure of the hydrogel [43,44].

### 3.5. Effect of Calcium Ions on Hydrogel Swelling Performance 

**Figure 4:**
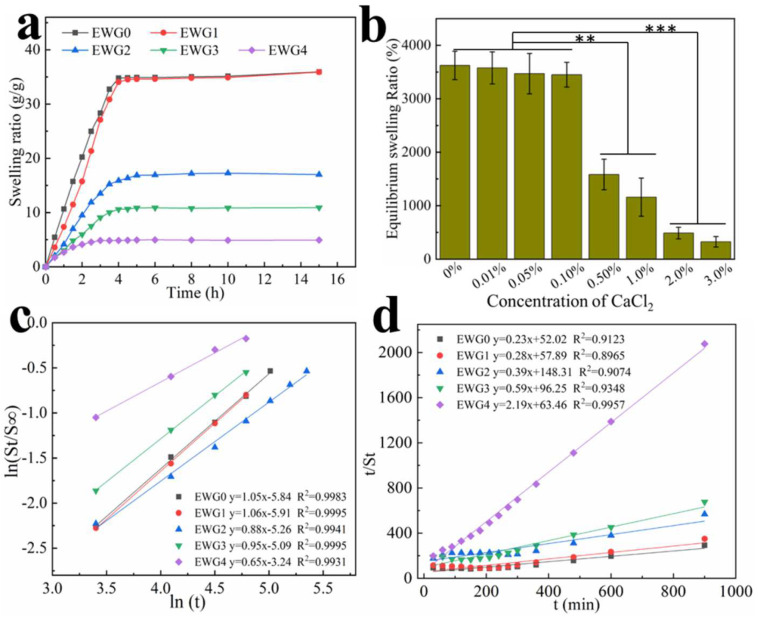
(**a**) Swelling kinetics of the hydrogels in distilled water at 37 °C; (**b**) equilibrium swelling ratio of the hydrogels in distilled water as a function of CaCl_2_ concentration. ** *p* < 0.01, *** *p* < 0.001; (**c**) plots of ln(S_t_/S_∞_) versus lnt; and (**d**) t/S_t_ versus t for the hydrogels.

The swelling performance of the hydrogels was strongly associated with hydrophilic groups and the pore network structure of the hydrogel, both of which are key to absorbing water. The swelling behavior of the hydrogel was tested in ultra-pure water. The results (Figure 4) show that the swelling rate increased rapidly with the extension of time and reached the swelling equilibrium after about 4 h. This pattern is common for hydrogels. Compared with the EWG0 group, EWG1, the hydrogel soaked in a low concentration of calcium ions (0.1%) showed a similar swelling rate, whereas EWG2, EWG3 and EWG4 showed gradual decreases with increasing concentration of calcium ions. The formation of the EWG0 hydrogel depended on the physical cross-linking with only weak binding between protein chains, giving water molecules easy access to the hydrogel interior and resulting in high expansibility. Moreover, it was found that the EWG0 hydrogel was gradually degraded and ruptured with extended soaking time, which was because the water molecules destroyed the three-dimensional structure with weak intermolecular forces [45]. It is well known that the degree of cross-linking directly affects the water absorption of a hydrogel [35]. When the egg white hydrogel was soaked in calcium chloride solution, calcium ions interacted with the amino acids of the protein chain to prompt the cross-linking and aggregation of the egg white protein and induce the formation of a tight three-dimensional network, resulting in significant decreases in water absorption and storage capacity of the hydrogels. Moreover, the cross-linked networks with tight structure could resist the destructive infiltration of moisture. The porosities of EWG0, EWG1, EWG2, EWG3 and EWG4 were 97.2 ± 2.34, 96.9 ± 3.16, 93.7 ± 4.24, 91.4 ± 4.94 and 82.4 ± 3.41, respectively (Figure S3). The porosities of the hydrogels decrease with increasing concentration of calcium ions, suggesting that the interaction between calcium ions and protein facilitate the cross-linking and aggregation of the protein chains, leading to the decrease of hydrogel porosity. It was found that the equilibrium swelling ratio of EWG0 was seven times higher than that of EWG4, indicating that the high concentration of calcium ions could effectively reduce the swelling capacity of the original egg white hydrogel. Therefore, it has great potential for preparing the sensitive double-layer hydrogel actuators using the EWG0 and EWG4 as the humidity responder and humidity inert layer, respectively (Figure S4) [46]. 

Swelling kinetic of the hydrogels is evaluated by Schott’s second-order diffusion kinetic model and Fickian diffusional kinetic model [47,48]. The swelling data achieved from the first 60% of the fractional water uptake are fitted with the following equation to determine water diffusion mechanism of hydrogel samples: ln(S_t_/S_∞_) = lnk + nlnt, where S_t_ and S_∞_ are the water uptake at time t and the equilibrium water uptake. The k parameter is a constant of the solvent-polymer system; the n parameter specifies the diffusion mechanism of water molecules. n < 0.5 indicates Fickian diffusion, 0.5 < n < 1 indicates non-Fickian diffusion and n = 1 indicates that the diffusion mechanism is case-II. Figure 4c shows the plots of ln(S_t_/S_∞_) versus lnt, the slopes and intercepts of the plotted lines could be used to calculate n and k. The values of n for EW0 and EWG1 are close to 1, indicating that the water diffusion mechanism in EWG0 and EWG1 is case-II (relaxation-controlled) transport. The values of n for EWG2, EWG3 and EWG4 are greater than 0.5, implying the water diffusion mechanisms are non-Fickian diffusion type. The water diffusion mechanism changes of the hydrogels are caused by the secondary cross-linking of the protein chain and the increased crosslinking density of hydrogels, which limit the protein chains relaxation and hinder the diffusion of water [49]. The Schott’s second-order diffusion kinetic model is used to get further information about the swelling rate: t/St = A + Bt, where $A=\frac{1}{K_{s}S_{\infty }^{2}}$ is the initial swelling rate of the hydrogel and K_s_ is the swelling rate constant, B = 1/S_∞_ is the converse of the equilibrium swelling. The plots of t/S_t_ versus t are plotted for the hydrogel samples (Figure 4d). The theoretical swelling equilibrium (shown in Table 2) of EWG0, EWG1, EWG2, EWG3 and EWG4 hydrogels are close to their corresponding experimental values. The swelling rate constants (K_s_) of EWG3 and EWG4 are higher than that of EWG0, suggesting that the hydrogels with high crosslinking density possess the faster swelling rate and reach the swelling equilibrium in a shorter time, which is consistent with the experimental results.

### 3.6. Effect of Calcium Ions on Hydrogel Texture 

**Figure 5:**
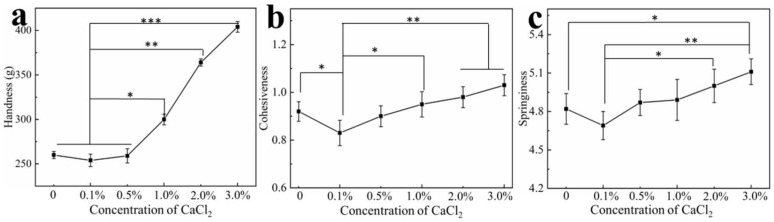
(**a**) Changes in hardness (**a**), cohesiveness (**b**) and springiness (**c**) of the hydrogels as a function of CaCl_2_ concentration. * *p* < 0.05, ** *p* < 0.01, *** *p* < 0.001.

Soaking the egg white hydrogel in different concentrations of calcium ions can change their microstructure, and, thereby, their properties. The textural properties of hardness, cohesiveness and springiness of the hydrogels were tested using a texture analyzer. As shown in Figure 5, the hardness of the hydrogels remained basically unchanged when the concentration of ions was less than 0.5% and when it was significantly enhanced as calcium ion concentration increased. Hardness is related to the structural strength of a hydrogel [50,51], and the overall structure of the protein is changed by the cross-linking of calcium ions with particular amino acids of adjacent peptides, resulting in the enhancement of the hydrogel hardness. Cohesiveness and springiness of the hydrogel were also affected by the addition of calcium. Previous studies [52,53] have shown that the conformation of proteins and polymerized protein chains are affected by divalent metal ions, leading to changes of the texture properties of protein hydrogels. In addition, cohesiveness and elasticity are influenced by the microstructure of the hydrogel. The SEM results showed that the introduction of calcium ions promoted a smaller three-dimensional pore structure and more compact microstructure of the hydrogel, which resulted in the enhancement of the cohesiveness and elasticity of the hydrogels. It could be found that the trends toward less hardness, cohesiveness and springiness of the hydrogels at low calcium concentration that might be because the calcium ions consumed hydroxyl ions, such that the three-dimensional network structure of the original hydrogel could not be maintained. As calcium concentration increased, the equilibrium between calcium and hydroxide ions was reached, and the interactions of the redundant calcium with the particular amino acids of the peptide chains gradually dominated and impacted the texture properties of the hydrogels.

### 3.7. Cytocompatibility 

**Figure 6:**
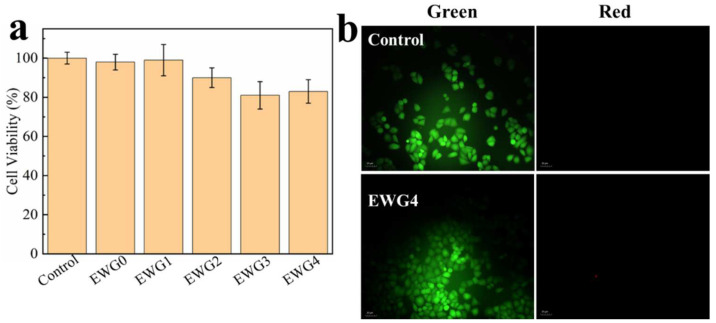
(**a**) Cell viability of HEK-293 cells on EWG0, EWG1, EWG2, EWG3 and EWG4 after 24 h culturing. (**b**) Live/dead staining florescent photographs of HEK-293 cells loaded with EWG4 for 48 h.

To investigate the potential of the hydrogels in biomedical applications (e.g., wound healing and bone tissue repair requiring calcium ions [54]), the hydrogels were co-cultured with HEK-293 cells for assessing the cytocompatibility and cell adhesion on the hydrogel. The MTT results (Figure 6a) showed that EWG0 and EWG1 showed cell survival rates similar to the control. With the increase of calcium chloride concentration, the cell survival rates of EWG2, EWG3 and EWG4 groups decreased slightly but remained above 80%, indicating that all hydrogels possessed cytocompatibility. Compared to the egg-white-/eggshell-based biomimetic hybrid hydrogels, the cells treated with EWG1 hydrogel for 24 h presented the similar proliferation rate [31], while the EWG2, EWG3 and EWG4 exhibited the lower proliferation rate, indicating that the high concentration calcium ions might be not advantageous to the cell proliferation. In addition, the live/dead cell staining assays (i.e., live cells stained fluorescent green, dead cells stained fluorescent red) were performed to study the cell adhesion and viability on the hydrogel surfaces. The results appear in Figure 6b. HEK-293 cells was able to survival normally and adhere to the EWG4 surface, demonstrating that the cross-linked hydrogels prepared with the highest calcium concentration were non-toxic, cytocompatible and adaptive for cell survival. In conclusion, calcium ion secondary cross-linked egg white gel showed excellent biocompatibility and biosafety. It should have great value for potential applications in the biomedical fields, particularly bone tissue engineering.

## 4. Conclusions

In summary, an egg white dual cross-linked hydrogel was prepared through the induction of sodium hydroxide and the secondary cross-linking of protein chains by calcium ions. Characteristics of the dual cross-linked hydrogel were remarkably affected by the concentrations of calcium ions. The incorporation of calcium ions could benefit thermal stability, swelling rate and texture of the hydrogels, while also reducing swelling capacity. Calcium ions could impact the secondary structure of polypeptide chains and interact with protein chains, leading to more compact microstructure formation of the hydrogels. Remarkably, the egg white dual cross-linked hydrogels exhibited biocompatibility and cell-surface adhesion in vitro, indicating the potential for biomedical application.

## Figures and Tables

**Table 1:** Effect of CaCl_2_ addition on the secondary structures of egg white protein.

Concentrations of CaCl_2_ Solution	Hydrogel Samples	Relative Content (%)			
		β-Sheets	Random Coil	α-Helices	β-Turns
0%	EWG0	23.56	14.93	14.56	46.96
0.1%	EWG1	34.54	12.35	11.41	41.71
0.5%	EWG2	33.21	13.55	13.34	39.91
1%	EWG3	36.38	14.54	15.11	33.98
2%	EWG4	21.98	31.12	15.81	31.08

**Table 2:** Second-order kinetic parameters for hydrogels.

Sample	EWG0	EWG1	EWG2	EWG3	EWG4
A	52.02	57.89	148.32	96.25	63.46
B	0.23	0.28	0.39	0.59	2.19
K_s_ × 10^−3^ (min^−1^)	1.08	1.39	1.09	3.68	75.83
S_∞_ (%)	3969.3	3501.9	1675.5	1756.4	492.5
ESR (%)	5463.1	4786.1	2657.8	2219.7	518.1

ESR: experimental swelling equilibrium value.

## Data Availability

The data presented in this study are available on request from the corresponding author.

## Supplementary Materials

The following supporting information can be downloaded at: https://www.mdpi.com/article/10.3390/polym14235116/s1, Figure S1. Normalized FT−IR spectra of hydrogels. Each peak represents a different secondary structure. (α-helices 1650–1658 cm^−1^, β-sheets 1640–1610 cm^−1^, β-turns 1700–1660 cm^−1^, random coils 1650–1640 cm^−1^). Figure S2. SEM images of the hydrogels. The scale bar is 5 µm. Figure S3. The porosities of the hydrogels. Figure S4. The photo of self-bending double layer hydrogel. The upper layer (white) is the hydrogel that soaked in calcium chloride, the lower layer (pale yellow) is the hydrogel that soaked without calcium chloride. The double layer hydrogel exhibited a smaller curvature that could be duo to the inapposite gel thickness.
