# Supplementary material for: Preparation and Properties of Egg White Dual Cross-Linked Hydrogel with Potential Application for Bone Tissue Engineering
Source: Polymers (Basel). 2022 Nov 24;14(23):5116. doi: 10.3390/polym14235116 (PMC9735576; doi:10.3390/polym14235116)
Supplement: Supplementary file 1 [file polymers-14-05116-s001.zip › polymers-1963685-supplementary.pdf]

Supporting Information

Preparation and properties of egg white dual cross-linked hydrogel with potential application for bone tissue engineering

Bingchao Duan, Minghui Yang, Quanchao Chao, Lan Wang, Lingli Zhang, Mengxing Gou, Yuling Li, Congjun Liu, Kui Lu**

School of Chemical Engineering and Food Science, Zhengzhou University of Technology, Zhengzhou, China

Correspondence to: Bingchao Duan, No.18, Yingcai Street, Huiji District, Zhengzhou, Henan Province, P. R. China.

*Correspondence to: Bingchao Duan.

E-mail: duanbc100@163.com

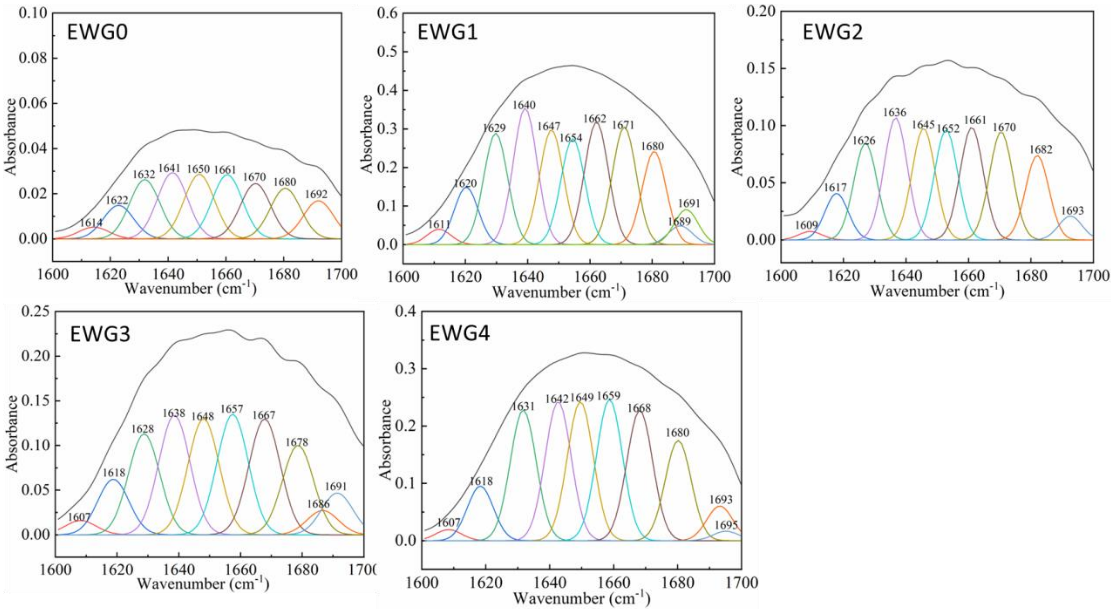

Figure S1. Normalized FT-IR spectra of hydrogels. Each peak represents a different secondary structure. (α-helices 1650-1658 cm^{-1}, β-sheets 1640-1610 cm^{-1}, β-turns 1700-1660 cm^{-1}, random coils 1650-1640 cm^{-1}).

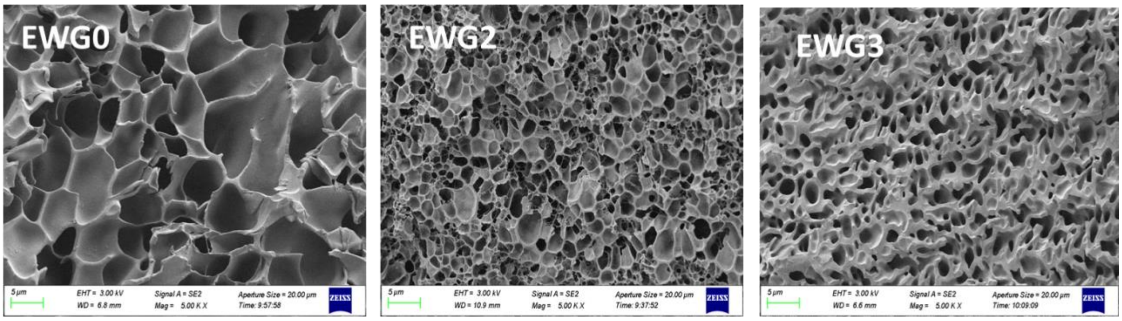

Figure S2. SEM images of the hydrogels. The scale bar is 5 μm.

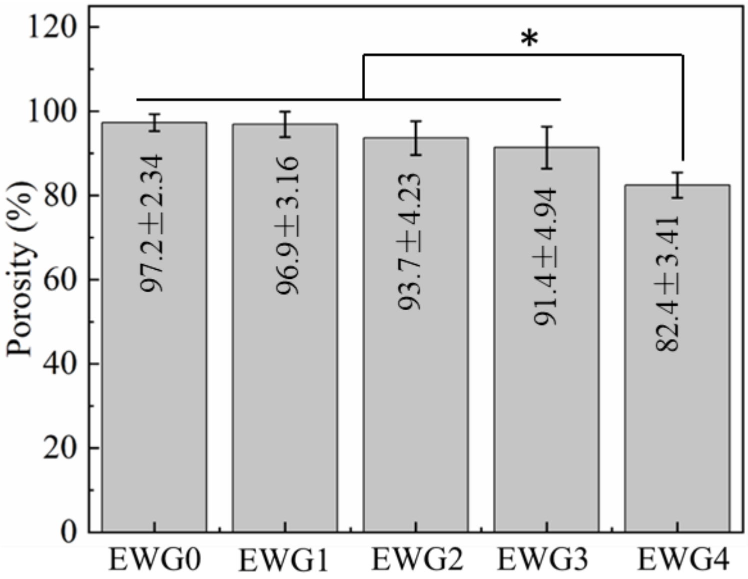

Figure S3. The porosities of the hydrogels.

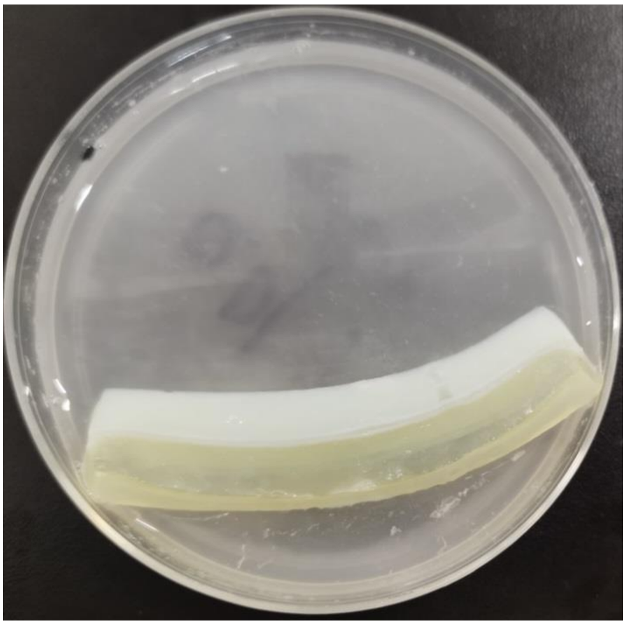

Figure S4. The photo of self-bending double layer hydrogel. The upper layer (white) is the hydrogel that soaked in calcium chloride, the lower layer (pale yellow) is the hydrogel that soaked without calcium chloride. The double layer hydrogel exhibited a smaller curvature that could be duo to the inapposite gel thickness.
